# Supplementary material for: E7 Oncogene HPV58 Variants Detected in Northeast Brazil: Genetic and Functional Analysis
Source: Microorganisms. 2023 Jul 27;11(8):1915. doi: 10.3390/microorganisms11081915 (PMC10458125; doi:10.3390/microorganisms11081915)
Supplement: Supplementary file 1 [file microorganisms-11-01915-s001.zip › microorganisms-2452435-supplementary.pdf]

Supplementary Table S1. Selective pressure estimates in the genomic region that include E7 gene of HPV58 based on log-likelihood, calculated by PAML program. The model that best fits the data are in bold. * means that values were determined from arithmetic mean.

ORF	Model	lnL	ω = dN/dS*	Parameters
**E7**	M0	-1442.267982	0.88348	ω = 0.88348
	M1	-1433.929983	0.35105	p_0 = 0.64895 (p_1 = 0.35105) ω_0 = 0.00000; ω_1 = 1.00000
	M2	-1417.952949	0.87940	p_0 = 0.95986; p_1 = 0.00000 (p_2 = 0.04014) ω_0 = 0.31914; ω_1 = 1.00000; ω_2 = 14.27708
	**M3**	**-1417.952637**	**0.87940**	**p_0 = 0.87683; p_1 = 0.08303 (p_2 = 0.04014) ω_0 = 0.31913; ω_1 = 0.31917; ω_2 = 14.27709**
	M7	-1434.108126	0.30000	p = 0.00500; q = 0.01158
	M8	-1417.953795	0.87960	p_0 = 0.95989 (p_1 = 0.04011) p = 46.49437; q = 99.00000; ω_s = 14.28357
